# Extracellular Microvesicle Production by Human Eosinophils Activated by “Inflammatory” Stimuli

**DOI:** 10.3389/fcell.2016.00117

**Published:** 2016-10-27

**Authors:** Praveen Akuthota, Lívia A. S. Carmo, Kennedy Bonjour, Ryann O. Murphy, Thiago P. Silva, Juliana P. Gamalier, Kelsey L. Capron, John Tigges, Vasilis Toxavidis, Virginia Camacho, Ionita Ghiran, Shigeharu Ueki, Peter F. Weller, Rossana C. N. Melo

**Affiliations:** ^1^Division of Allergy and Inflammation, Department of Medicine, Beth Israel Deaconess Medical Center, Harvard Medical SchoolBoston, MA, USA; ^2^Division of Pulmonary and Critical Care Medicine, Department of Medicine, University of California San DiegoLa Jolla, CA, USA; ^3^Laboratory of Cellular Biology, Department of Biology, Institute of Biological Sciences (ICB), Federal University of Juiz de ForaJuiz de Fora, Brazil; ^4^Flow Cytometry Core, Department of Medicine, Beth Israel Deaconess Medical Center, Harvard Medical SchoolBoston, MA, USA; ^5^Department of General Internal Medicine and Clinical Laboratory Medicine, Akita University Graduate School of MedicineAkita, Japan

**Keywords:** cell secretion, inflammation, CCL11 (eotaxin-1), tumor necrosis factor alpha (TNF-α), tetraspanins, CD63, CD9, transmission electron microscopy (TEM)

## Abstract

A key function of human eosinophils is to secrete cytokines, chemokines and cationic proteins, trafficking, and releasing these mediators for roles in inflammation and other immune responses. Eosinophil activation leads to secretion of pre-synthesized granule-stored mediators through different mechanisms, but the ability of eosinophils to secrete extracellular vesicles (EVs), very small vesicles with preserved membrane topology, is still poorly understood. In the present work, we sought to identify and characterize EVs released from human eosinophils during different conditions: after a culturing period or after isolation and stimulation with inflammatory stimuli, which are known to induce eosinophil activation and secretion: CCL11 (eotaxin-1) and tumor necrosis factor alpha (TNF-α). EV production was investigated by nanoscale flow cytometry, conventional transmission electron microscopy (TEM) and pre-embedding immunonanogold EM. The tetraspanins CD63 and CD9 were used as EV biomarkers for both flow cytometry and ultrastructural immunolabeling. Nanoscale flow cytometry showed that human eosinophils produce EVs in culture and that a population of EVs expressed detectable CD9, while CD63 was not consistently detected. When eosinophils were stimulated immediately after isolation and analyzed by TEM, EVs were clearly identified as microvesicles (MVs) outwardly budding off the plasma membrane. Both CCL11 and TNF-α induced significant increases of MVs compared to unstimulated cells. TNF-α induced amplified release of MVs more than CCL11. Eosinophil MV diameters varied from 20 to 1000 nm. Immunonanogold EM revealed clear immunolabeling for CD63 and CD9 on eosinophil MVs, although not all MVs were labeled. Altogether, we identified, for the first time, that human eosinophils secrete MVs and that this production increases in response to inflammatory stimuli. This is important to understand the complex secretory activities of eosinophils underlying immune responses. The contribution of the eosinophil-derived MVs to the regulation of immune responses awaits further investigation.

## Introduction

Eosinophils, leukocytes of the innate immune system that are involved in the pathogenesis of asthma, allergies, and other diseases as well as other ongoing homeostatic roles in tissues, have a remarkable ability to secrete specific proteins in response to inflammatory stimuli. A plethora of mediators are stored as preformed molecules within eosinophil specific (secretory) granules, the singular granule population in the cytoplasm of these cells such as distinct cationic proteins and cytokines (reviewed in Spencer et al., 2014).

Some major mechanisms leading to secretion of granule-derived immune mediators have been well-characterized in human eosinophils. In response to cell activation, granules can fuse with the plasma membrane in order to secrete their contents, but the most frequent mechanism for the delivery of eosinophil mediators involve vesicular carriers, which recruit cargos directly from secretory granules, a secretory process termed piecemeal degranulation (reviewed in Melo and Weller, 2010; Melo et al., 2013a; Spencer et al., 2014). While the study of eosinophil degranulation processes has received great attention in the last decade, the ability of eosinophils to secrete membrane vesicles, collectively termed extracellular vesicles (EVs), remains to be explored.

Various names, including exosomes and microvesicles (MVs)/microparticles, have been given to secreted EVs. While the term exosomes is used for referring to a population of EVs, which are released from cells when multivesicular bodies (MVBs) fuse with the plasma membrane, the term MVs has been generally used for EVs formed by budding and shedding of the plasma membrane (reviewed in van der Pol et al., 2012; Twu and Johnson, 2014; Lawson et al., 2016). Recently, it was demonstrated that human eosinophils secrete exosomes in culture cell conditions and that this type of EV is increased in asthmatic patients, which links EVs with eosinophil activation (Mazzeo et al., 2015). However, the functions of EVs secreted by immune cells during inflammatory responses are still poorly understood. It is believed that these vesicles can act as carriers of cell-cell communication mediators such as cytokines and lipid mediators, and potentially contribute to inflammation (reviewed in Buzas et al., 2014). Moreover, a potential immunomodulatory role for treating or preventing inflammatory disorders has been attributed to EVs (Buzas et al., 2014).

EVs secreted by cells can be detected by nanoscale flow cytometric methods, which identify and sort submicron particles (Danielson et al., 2016) and transmission electron microscopy (TEM), which enables unambiguous visualization of EVs (reviewed in Lawson et al., 2016). EM is thus considered an essential technique to characterize EVs and to distinguish them from non-membranous particles of similar size, as endorsed by the International Society for EVs in an effort to provide minimal requirements for EV definition (Lötvall et al., 2014).

**Figure 1:**
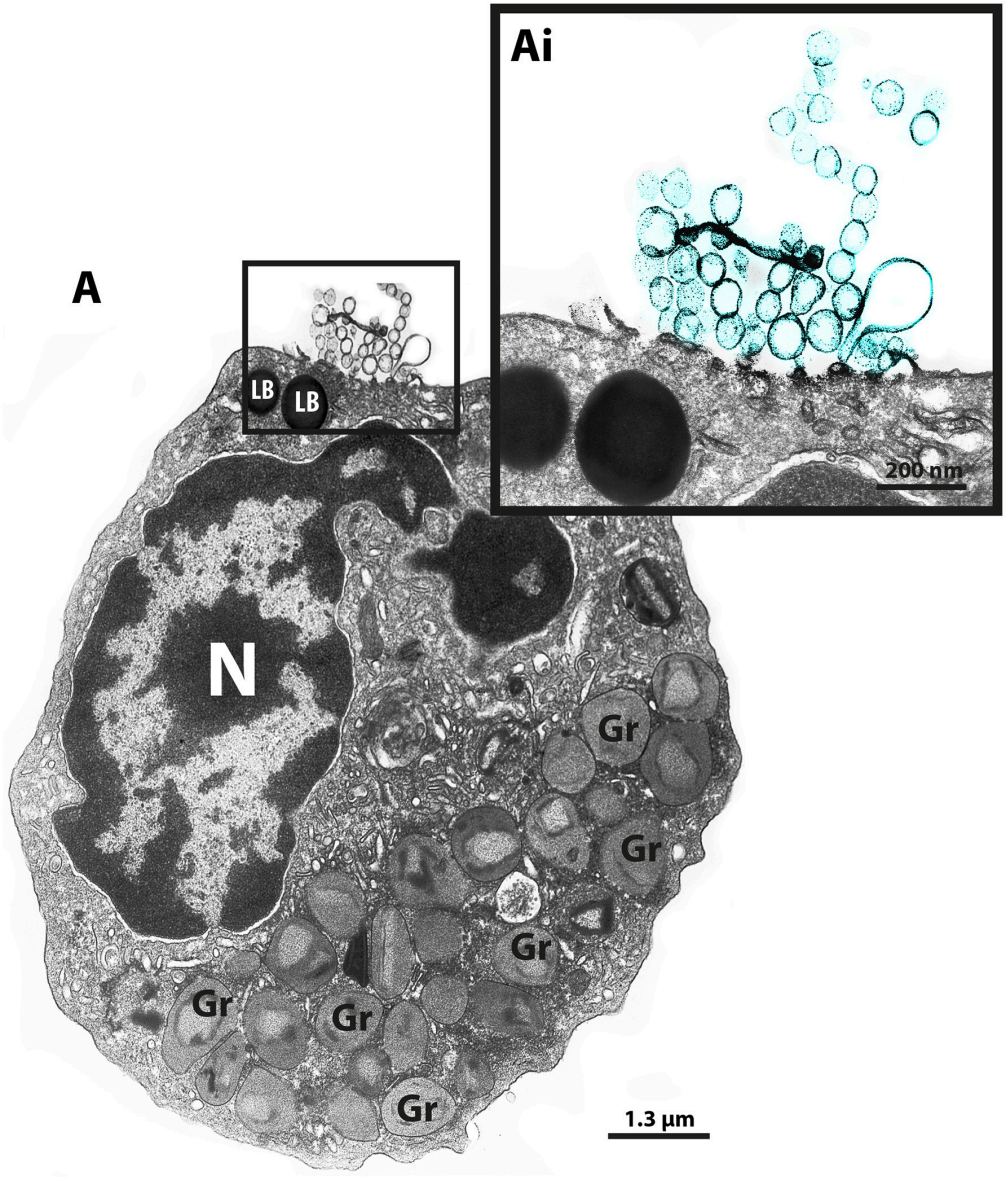
**TEM reveals production of MVs by human eosinophils**. **(A)** A representative micrograph of a human eosinophil documents several MVs (highlighted in blue in **Ai**) budding directly from the plasma membrane. Eosinophil specific granules (Gr), the singular population of secretory granules in the cytoplasm, show lucencies in their granule cores, indicative of cell activation. Two lipid bodies (LB), which typically appear as very electron-dense organelles in eosinophils, are seen in the cell periphery. Eosinophils were isolated from the peripheral blood from healthy donors by negative selection, kept in medium during 1 h, immediately fixed while still in suspension and processed for conventional TEM. N, nucleus.

Our group has been using different EM techniques, including conventional TEM and immunonanogold EM, to understand mechanisms of vesicular trafficking and release of immune mediators from human eosinophils activated by inflammatory stimuli (Melo et al., 2005a,b, 2008a, 2009, 2010; Spencer et al., 2006; Carmo et al., 2015). By studying the ultrastructure of human eosinophils isolated from the peripheral blood, we noticed the presence of EVs budding from the cell surface when the cells were kept alive in medium (Figure 1).

In the present work, we sought to identify and characterize EVs released from human eosinophils during different conditions: in culture and after stimulation with two distinct agonist “inflammatory” stimuli, which are known to induce eosinophil activation and secretion: the chemokine, CCL11 (eotaxin-1), and the cytokine, tumor necrosis factor alpha (TNF-α; Egesten et al., 1998; Bandeira-Melo et al., 2001, 2003; Liu et al., 2007; Spencer et al., 2009). In recent work, we showed that these stimuli trigger increased formation of intracellular transport vesicles in association with distinct processes of eosinophil secretion (Carmo et al., 2016). We wondered if both stimuli are also able to influence the biogenesis of EVs. By performing a comprehensive study, using nanoscale flow cytometry, conventional TEM and immunonanogold labeling for CD63 and CD9, we demonstrate that human eosinophils produce EVs, which were clearly characterized as MVs, and that this production is increased in response to both CCL11 and TNF-α, identifying eosinophil EV genesis as a secretory mechanism with eosinophil-mediated immune responses.

## Materials and methods

### Eosinophil isolation, stimulation, and viability

Granulocytes were isolated from peripheral blood of allergic or healthy donors. Eosinophils were enriched and purified by negative selection as previously described (StemSep^TM^, StemCell Technologies, Seattle WA; Miltenyi Biotec, Auburn, CA; Bandeira-Melo et al., 2000; Akuthota et al., 2014). The hypotonic red blood cell (RBC) lysis was omitted to avoid any potential for RBC lysis to affect eosinophil function. Eosinophil viability and purity were >99% as determined by ethidium bromide (Molecular Probes, Life Technologies, Carlsbad, CA) incorporation and cytocentrifuged smears stained with HEMA 3 stain kit (Fisher Scientific, Medford, MA), respectively. Purified eosinophils (10^6^ cells/mL) were stimulated with TNF-α (200 ng/mL; R&D Systems, Minneapolis, MN) or recombinant human CCL11 (100 ng/mL; R&D Systems), in RPMI-1640 medium plus 0.1% ovalbumin (OVA; Sigma, St. Louis, MO, USA), or medium alone at 37°C, for 1 h as before (Carmo et al., 2016).

### Ethics statement

Written informed consent was obtained from donors in accordance with the Declaration of Helsinki, and Institutional Review Board (IRB) approval was obtained from the Beth Israel Deaconess Medical Center Committee on Clinical Investigation (Boston, MA, USA).

### Antibody reagents

Mouse anti-human IgG_1_ CD63 (clone H5C6, catalog number 556019, 5 μg/mL, BD-Pharmingen, San Diego, CA), mouse anti-human CD9 (clone 209306; R&D Systems, 10 μg/mL, Minneapolis, MN) and irrelevant isotype control monoclonal antibodies (mAbs) were used for electron microscopy immunodetection studies. The secondary Ab for immunoEM was an affinity-purified goat anti-mouse Fab fragment conjugated to 1.4 nm gold particles (1:100, Nanogold, Nanoprobes, Stony Brook, NY). FITC-conjugated mouse anti-human IgG_1_ CD63 (clone H5C6, Biolegend, San Diego, CA), FITC-conjugated mouse anti-human IgG_1_ CD9 (clone HI9a, Biolegend), and irrelevant FITC-conjugated isotype control antibodies were used for nanoscale flow cytometry or regular flow cytometry.

### Nanoscale flow cytometry

Human eosinophils were incubated for 4 days in RPMI-1640 with 5% FBS with 10 ng/mL IL-5 and 1 ng/mL of GM-CSF to allow for EV accumulation in the culture supernatant. Due to the presence of FBS, culture medium was depleted of EVs by ultracentrifugation prior to use. Supernatants were depleted of eosinophils and debris with successive centrifugation at 300 × g, 5600 × g, and 11,000 × g. EVs were then isolated by ultracentrifugation at 100,000 × g for 1 h. EV-depleted culture medium without cells present subjected to the same protocol served as a negative control. Prior to nanoscale flow cytometry, some samples were incubated with FITC-conjugated anti-CD9 antibody or FITC-conjugated anti-CD63 antibody. Nanoscale flow cytometry was performed as previously described using a Beckman Coulter MoFlo AstriosEQ modified to optimize detection of small particles down to 200 nm in diameter (Danielson et al., 2016). Control Latex Beads were obtained from Beckman Coulter. Electronic noise was gated out during analysis using the signal generated by phosphate buffered saline alone as a reference.

### Flow cytometry for CD63 in entire cells

For CD63 detection in human eosinophils by regular flow cytometry, cells were incubated 1:25 in relevant antibody or isotype control for 25 min at 4°C. Flow cytometry for CD63 was performed using a BD Accuri Flow Cytometer. Data were analyzed using FlowJo (TreeStar, Ashland, OR, USA).

### Conventional TEM

For conventional TEM, isolated eosinophils were prepared as before (Melo et al., 2005a, 2009). Cells were fixed in a mixture of freshly prepared aldehydes [1% paraformaldehyde (PFO) and 1.25% glutaraldehyde] in 0.1 M sodium cacodylate buffer (final concentration) for 1 h at RT, embedded in 2% agar and kept at 4°C for further processing. Agar pellets containing eosinophils were post-fixed in 1% osmium tetroxide in sym-collidine buffer, pH 7.4, for 2 h at RT. After washing with sodium maleate buffer, pH 5.2, pellets were stained en bloc in 2% uranyl acetate in 0.05 M sodium maleate buffer, pH 6.0 for 2 h at RT and washed in the same buffer as before prior to dehydration in graded ethanols and infiltration and embedding with a propylene oxide-Epon sequence (Eponate 12 Resin; Ted Pella, Redding, CA). Sections were mounted on uncoated 200-mesh copper grids (Ted Pella) before staining with lead citrate and viewed with a transmission electron microscope (CM 10; Philips, Eindhoven, The Netherlands) at 60 KV.

### Cell preparation for immunonanogold EM

For immunoEM, purified eosinophils were immediately fixed in fresh 4% PFO in PBS, pH 7.4 (Melo et al., 2014). Cells were fixed for 30 min at RT, washed in PBS and centrifuged at 1500 g for 1 min. Samples were then resuspended in molten 2% agar in PBS and quickly recentrifuged. Pellets were immersed in 30% sucrose in PBS overnight at 4°C, embedded in OCT compound (Miles, Elkhart, IN), and stored in −180°C liquid nitrogen for subsequent use.

### Pre-embedding immunonanogold EM

As detailed before (Melo et al., 2005b, 2009; Dias et al., 2014), pre-embedding immunolabeling was carried out before standard EM processing (post-fixation, dehydration, infiltration, resin embedding and resin sectioning). All labeling steps were carried out at RT as before (Melo et al., 2014) as follows: (a) one wash in 0.02 M PBS, pH 7.6, 5 min; (b) immersion in 50 mM glycine in 0.02 M PBS, pH 7.4, 10 min; (c) incubation in a mixture of PBS and BSA (PBS-BSA buffer; 0.02 M PBS plus 1% BSA) containing 0.1% gelatin (20 min) followed by PBS-BSA plus 10% normal goat serum (NGS; 30 min)—(this step is crucial to block non-specific Ab binding sites); (d) incubation with primary Ab (1 h); (e) blocking with PBS-BSA plus NGS (30 min); (f) incubation with secondary Ab (1 h); (g) washing in PBS-BSA (three times of 5 min each); (h) post-fixation in 1% glutaraldehyde (10 min); (i) five washings in distilled water; (j) incubation with *HQ silver enhancement* kit (Nanoprobes) in a dark room according to the manufacturer's instructions (10 min). This step enables a nucleation of silver ions around gold particles. These ions precipitate as silver metal and the particles grow in size facilitating observation under TEM); (k) three washings in distilled water; (l) immersion in freshly prepared 5% sodium thiosulfate (5 min); (m) post-fixation with 1% osmium tetroxide in distilled water (10 min); (n) staining with 2% uranyl acetate in distilled water (5 min); (o) embedding in Eponate (Eponate 12 Resin; Ted Pella); (p) after polymerization at 60°C for 16 h, embedding was performed by inverting eponate-filled plastic capsules over the slide-attached tissue sections; and (q) separation of eponate blocks from glass slides by brief immersion in liquid nitrogen. Thin sections were cut using a diamond knife on an ultramicrotome (Leica). Sections were mounted on uncoated 200-mesh copper grids (Ted Pella) before staining with lead citrate and viewed with a transmission electron microscope (CM 10; Philips) at 60 kV. Two controls were performed: (1) primary Ab was replaced by an irrelevant Ab, and (2) primary Ab was omitted. Electron micrographs were randomly taken at different magnifications to study the entire cell profile and subcellular features.

### Quantitative EM analysis

For quantification studies by conventional TEM (enumeration of the total number of EVs and MVBs), electron micrographs of cell sections were randomly taken from unstimulated and stimulated eosinophils. Electron micrographs were taken by an operator blind to EV identification. A total of 110 electron micrographs (39 from unstimulated, 37 from CCL11- and 34 from TNF-α-stimulated eosinophils) and 516 EVs (55 from unstimulated, 187 from CCL11- and 274 from TNF-α-stimulated eosinophils) were counted. Then, the diameters of EVs were measured and grouped in different ranges (20–100, 100–200, 200–300, 300–1000 nm). The presence of typical MVBs was investigated in all electron micrographs. These analyses were done in clear cross-cell sections exhibiting the entire eosinophil cell profile, intact plasma membranes and nuclei. EVs were morphologically defined as intact, small round vesicles, delimited by a membrane unit, which is seen by TEM as a typical trilaminar structure, in process of outward budding from the plasma membrane or closely associated with the cell surface.

For immunonanogold EM studies, a total of 69 electron micrographs randomly taken from unstimulated and stimulated eosinophils were evaluated for CD63 and CD9 labeling. These analyses were done in clear cross-cell sections exhibiting the entire eosinophil cell profile, intact plasma membranes, and nuclei.

All quantitative studies were performed using the *Image J* software (National Institutes of Health, Bethesda, MD).

### Protein electrophoresis of EVs

For protein electrophoresis of EVs, culture supernatants of primary human eosinophils were collected and centrifuged as they were for nanoscale flow cytometry. Culture supernatant from an eosinophilic leukemia cell line (Eol-1, Sigma-Aldrich) was also collected and centrifuged. Lithium dodecyl sulfate sample buffer (4X; Invitrogen) and sample reducing agent (10X) (Invitrogen) were added at final 1X concentrations. Samples, first heated for 7.5 min at 95°C, were run on a 4–12% Bis-Tris gel. Silver staining was then performed of polyacrylamide gel (Thermo Scientific, Rockford, IL, USA) were used to develop membranes.

### Annexin V analysis by confocal microscopy and flow cytometry

To detect exposed phosphatidylserine, cells were stained with annexin V (Gonzalez-Cano et al., 2010). Freshly isolated human eosinophils were resuspended in 5% FBS RPMI-1640 medium (10^6^ cells/mL) and stimulated, as above, at 37°C in 5% CO2 incubator. Annexin V-FITC (Medical and Biological Laboratories, Nagoya, Japan) was then added to the culture medium (1:20) and cells were viewed without washing or fixing in an imaging chamber (Zell-kontakt, Nörten-Hardenberg, Germany). The confocal microscopic and differential interference contrast (DIC) images were captured using a laser scanning confocal microscope with incubation chamber (100x objective, Carl Zeiss LSM780, Jena, Germany). For flow cytometry, annexin V-FITC stained cells were measured using a flow cytometer (Cytomics FC500, Beckman Coulter, Fullerton, CA, USA). Data were analyzed by Flowjo software.

### Tunel assay

Eosinophils stimulated as described were fixed with 4% paraformaldehyde and stained using MEBSTAIN Apoptosis TUNEL Kit (Medial Biological Laboratories, Nagoya, Japan) according to the manufacturer's instruction. Images were captured using a fluorescence microscope (40x objective, Leica DMI 4000B, Wetzlar, Germany).

### Statistical analyses

Comparison between groups was analyzed using Kruskal Wallis test followed by Dunn's test to adjust for multiple comparisons, as appropriate. The significance level was set at *P* < 0.05. All tests and graphs were performed with software Prism 6.0.1 (GraphPad software, San Diego, CA). Data are expressed as means ± SEM.

## Results

### Human eosinophils release EVs

Over the last decade, our research group has been studying the ultrastructure of human eosinophils during different conditions. Our EM methodology, primarily developed for studying human eosinophils isolated from the peripheral blood, includes prompt aldehyde fixation while the cells are still in suspension, which is important to optimal cell preservation and to capture specific biological events in response to varied stimuli (Melo et al., 2005a, 2013b). Thus, cells kept alive in suspension either unstimulated or agonist stimulated are immediately fixed after a determined time, before any subsequent centrifugation procedure, which could interfere with the cell morphology. While examining resulting electron micrographs from different experiments, we occasionally noticed clear shedding of small vesicles delimited by a typical phospholipid bilayer from the eosinophil surface (Figure 1).

**Figure 2:**
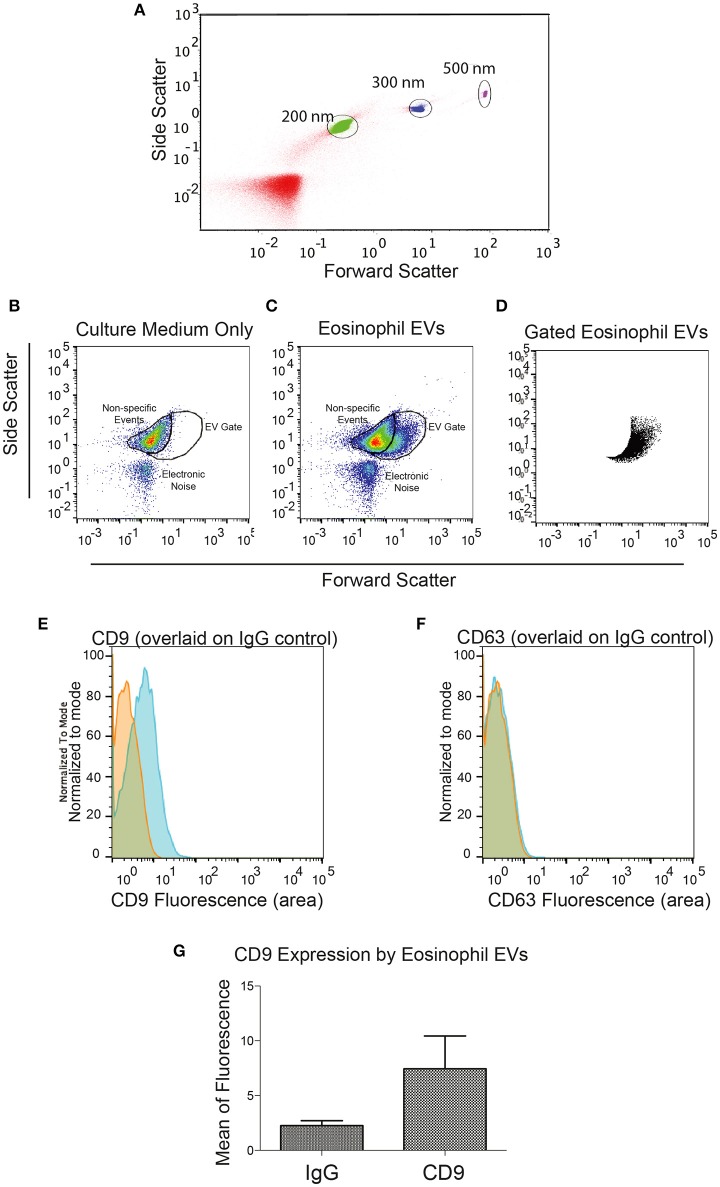
**Identification of Eosinophil EVs by Nanoscale Flow Cytometry. (A)** The ability of the AstriosEQ to discriminate sub-micrometer particles is shown using a mixture of Control Latex Beads (200, 300, and 500 nm). **(B)** Flow cytometry of RPMI with FBS (EV-depleted) alone showing non-specific events and electronic noise. Forward scatter and side scatter plotted. **(C)** Flow cytometry of human eosinophil EVs in RPMI with FBS (EV-depleted). Gate drawn around EV signal (FSC and SSC plotted). **(D)** Gated eosinophil EVs. **(E)** Human eosinophil EVs CD9 expression (blue) overlaid on IgG control (red). **(F)** Human eosinophil CD63 expression (blue) overlaid on IgG control (red) shows minimal detection of CD63 by nanoscale flow cytometry. Representative of four experiments from four individual donors. **(G)** Mean CD9 fluorescence (*p* = 0.13, paired *t*-test). Panels **(B–G)** are representative of four independent experiments from four individual human donors.

We then decided to investigate whether eosinophils kept in culture were able to release EVs. Eosinophils isolated from the peripheral blood of healthy patients were incubated for 4 days in culture to allow EV accumulation in the culture supernatant. EVs isolated by ultracentrifugation were evaluated by nanoscale flow cytometry. Using standard latex beads, we first confirmed the ability of the nanoscale flow cytometry approach to discriminate small particles down to a size of 200 nm and lower (Figure 2A). After gating out electronic noise, EVs derived from human eosinophil cultures were identified (Figures 2B–D). On staining EVs with FITC-conjugated anti-CD9 or anti-CD63 antibodies, we found that EVs, that were identifiable and evaluated by nanoscale flow cytometry, had readily detectable CD9 (Figure 2E). A band at 25 kD, consistent with the presence of CD9 in these vesicles, was also detected by protein electrophoresis (Supplementary Figure 1). CD63 expression by eosinophil EVs was not detectable with nanoscale flow cytometry (Figure 2F). All nanoscale flow cytometry results were representative of four individual experiments from four individual normal donors (Figure 2). Positive controls for CD63 are shown in Supplementary Figure 2.

### EV production by human eosinophils increases in response to inflammatory stimuli

**Figure 3:**
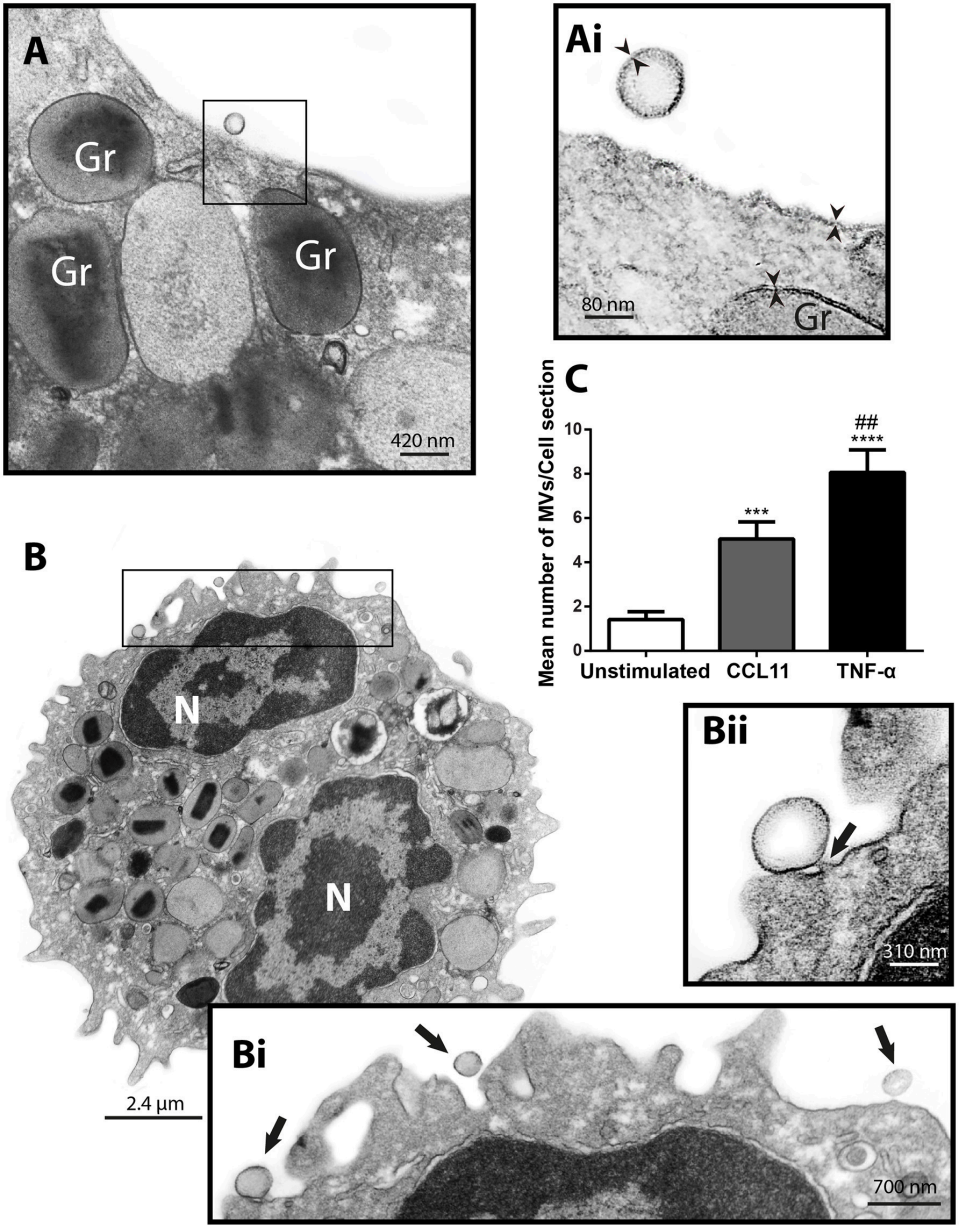
**CCL11 and TNF-α stimulation induce release of MVs by human eosinophils**. **(A, Ai, B, Bi, Bii)** MVs are seen at the surface of both unstimulated **(A)** and CCL11-stimulated **(B)** human eosinophils. Note in high magnification **(Ai)** that the phospholipid bilayer membrane, which is seen by TEM as a trilaminar structure (arrowheads), is observed around the EVs, plasma membrane and secretory granule (Gr) delimiting membrane. **(Bii)** Shows in high magnification a MV in final process of detaching from the plasma membrane (arrow). **(C)** Significant increases in numbers of MVs occurred after stimulation with CCL11 or TNF-α. Eosinophils were isolated from the peripheral blood by negative selection, stimulated for 1 h, immediately fixed and processed for conventional TEM. Counts were derived from three experiments with a total of 516 MVs counted in 110 electron micrographs randomly taken and showing the entire cell profile and nucleus (N). Data represent mean ± S.E.M. ^***^P < 0.002 (CCL11 vs. unstimulated); ^****^P < 0.0001 (TNF-α vs. unstimulated); ^##^*P* < 0.02 (TNF-α vs. CCL11).

Next, to study the phenomenon of vesicle release and detect EVs at the cell surface, we stimulated freshly isolated eosinophils from normal donors during 1 h with CCL11 or TNF-α, at concentrations previously documented to induce secretion, or medium alone (Carmo et al., 2016) and immediately processed for conventional TEM. Then, electron micrographs randomly taken from the thin sections by an operator blind to EV identification and showing the entire cell profile and intact plasma membrane were carefully examined. First, conventional TEM revealed that EVs appeared mostly as MVs in both unstimulated (Figure 3A) and stimulated (Figure 3B) cells, that is, shedding directly from the plasma membrane. Typical MVs, delimited by a phospholipid membrane, were seen in progressive outward budding of the plasma membrane (Figure 3B) and/or completely released at cell surface (Figures 3A,B).

**Figure 4:**
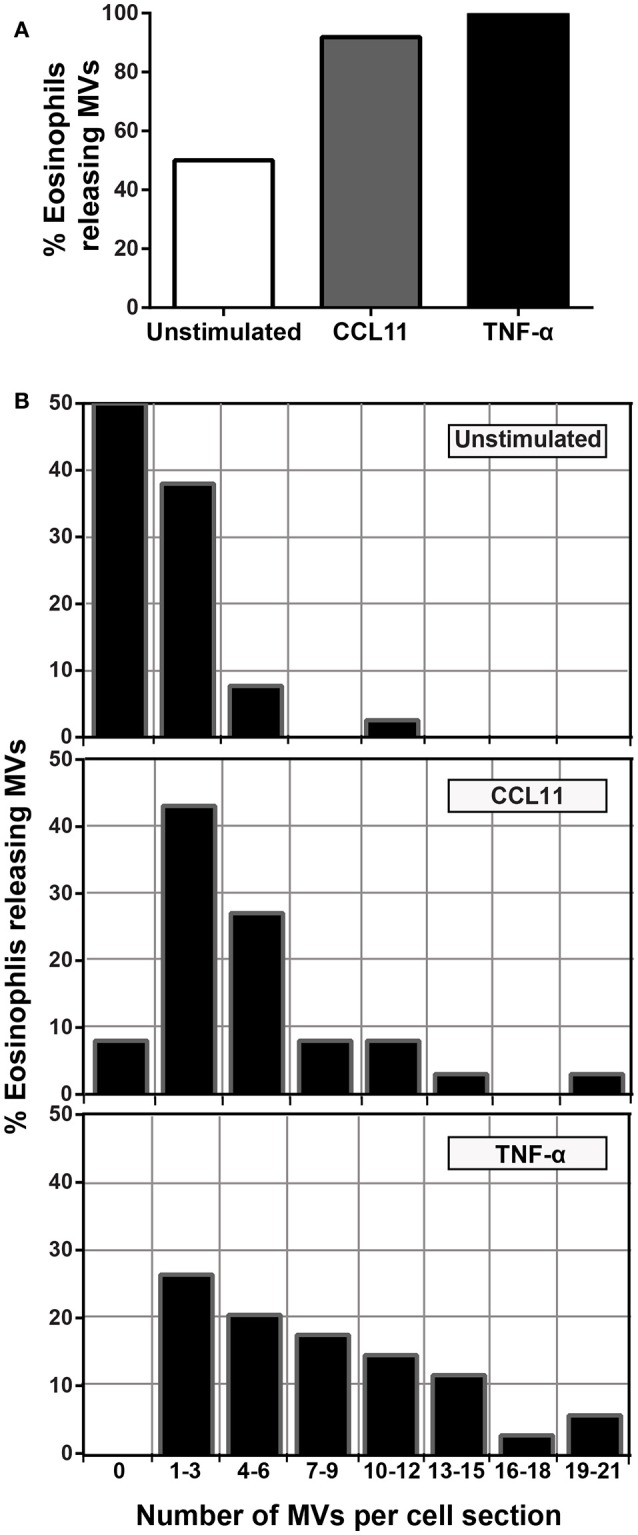
**Proportion of eosinophils releasing MVs. (A)** While just 50% of unstimulated cells produced MVs, 90 and 100% of eosinophils formed MVs in CCL11 and TNF-α-stimulated eosinophils, respectively. **(B)** Heterogeneity of cell responses in unstimulated and stimulated eosinophils. In unstimulated cells, most MV-producing cells (30%) released 1–3 MVs/cell section whereas ~70% of cells produced 1–9 MVs and 4–21 MVs/cell section in response to CCL11 and TNF-α stimulation, respectively. Counts were derived from three experiments with a total of 516 MVs counted in 110 electron micrographs randomly taken and showing the entire cell profile and nucleus.

To quantify the number of MVs from each experimental group, eosinophil sections showing the entire cell profile and nucleus were evaluated (*n* = 110 cells), and a total of 516 MVs were counted. Eosinophil activation led to a significant increase of MV production compared to unstimulated cells (Figure 3C). Quantitative EM revealed that while unstimulated cells had 1.4 ± 0.4 MVs/cell section, CCL11- and TNF-α-stimulated cells showed 5.0 ± 0.8 (*P* = 0.0014) and 8.0 ± 1.0 (*P* < 0.0001) MVs/cell section (mean ± S.E.M), respectively (Figure 3C), corresponding to an increase of 360% (CCL11) and 570% (TNF-α). TNF-α induced a significant increase in the release of MVs compared to CCL11 (*P* = 0.0116; Figure 3C). Moreover, our quantitative analyses showed that just 50% of unstimulated cells produced MVs whereas 90 and 100% of eosinophils formed MVs in CCL11- and TNF-α-stimulated groups, respectively (Figure 4A). Moreover, by scoring the number of MVs, we found that in unstimulated cells, most MV-producing cells (30%), released 1–3 MVs/cell section whereas ~70% of cells produced 1–9 MVs and 4–21 MVs/cell section in response to CCL11 and TNF-α stimulation, respectively (Figure 4B).

**Figure 5:**
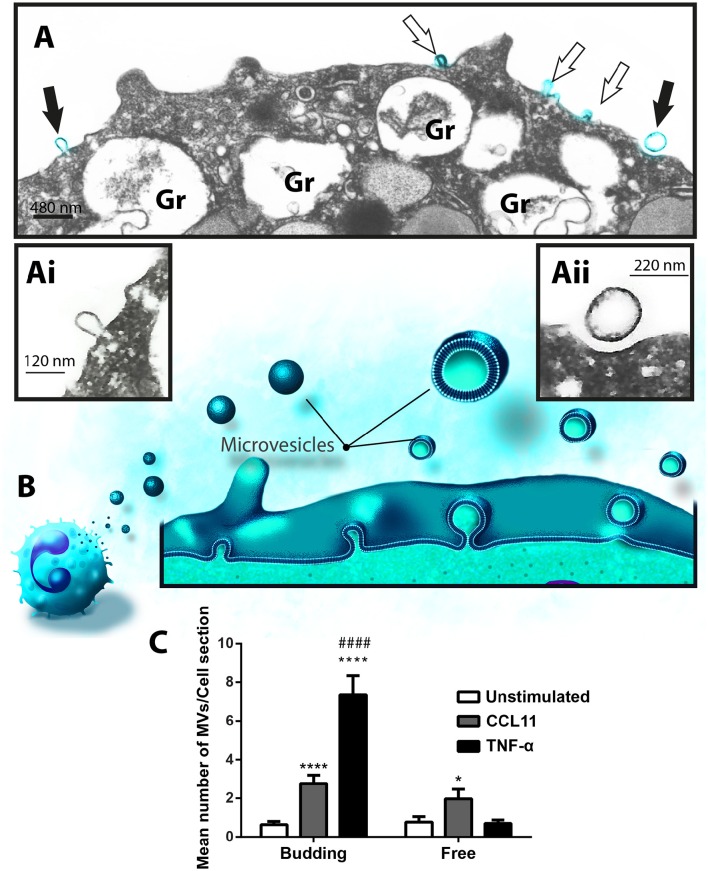
**Differential release of nascent MVs by human activated eosinophils. (A)** A representative electron micrograph of a TNF-α-stimulated eosinophil shows MVs in different steps of budding at cell surface (highlighted in blue, arrows) and secretory granules (Gr) exhibiting content losses in the cytoplasm. The MVs indicated by the black arrows are seen in high magnification in **(Ai)** and **(Aii)**. Note, that while **(Ai)** shows a MV in process of outward budding; **(Aii)** shows a free MV, completely detached from the plasma membrane. **(B)** Illustration depicting the process of MV formation in human eosinophils as observed in the present work. **(C)** Significant increases in numbers of budding MVs occurred after stimulation with CCL11 or TNF-α compared to unstimulated cells (^****^P < 0.0001). TNF-α elicited higher numbers of MVs in process of budding compared to the CCL11 group (^####^*P* < 0.0001). Increase in numbers of free vesicles occurred after stimulation with CCL11 compared to unstimulated cells (^*^P = 0.020). Eosinophils were isolated from the peripheral blood by negative selection, stimulated for 1 h, immediately fixed in suspension and processed for conventional TEM. Counts were derived from three experiments, with a total of 516 MVs counted in 110 electron micrographs randomly taken and showing the entire cell profile and nucleus (N).

Formation of MVs is a dynamic process and therefore these vesicles may be observed by TEM in different stages of budding from the plasma membrane or free at the cell surface (Figures 3A,B, 5A). Because our TEM studies have clearly captured this process as illustrated in Figure 5B, we next wondered if there was any difference in the numbers of budding/free MVs per treatment condition. Indeed, the numbers of budding MVs were significantly higher in stimulated compared to unstimulated cells [7.35 ± 0.98 for TNF-α- and 2.75 ± 0.44 for CCL11-stimulated groups vs. 0.64 ± 0.16 for unstimulated cells; MVs/cell section (mean ± S.E.M); *P* < 0.0001; Figure 5C]. Interestingly, the number of MVs in different degrees of budding was higher in TNF-α-stimulated compared to CCL11-stimulated cells (Figure 5C; *P* < 0.0001). Altogether, our findings reveal that two eosinophil agonist “inflammatory” stimuli induce vesiculation and that this event is more prominent in TNF-α- compared to CCL11-stimulated cells, since the number of nascent MVs was significantly higher in the TNF-α group (Figure 5C). Of note, the presence of MVBs was detected within eosinophils from all groups (Supplementary Figure 3). However, we did not find evidence for fusion of them with the plasma membrane and resulting exososome release (Supplementary Figure 3).

### Ultrastructural characterization of eosinophil-secreted MVs

**Figure 6:**
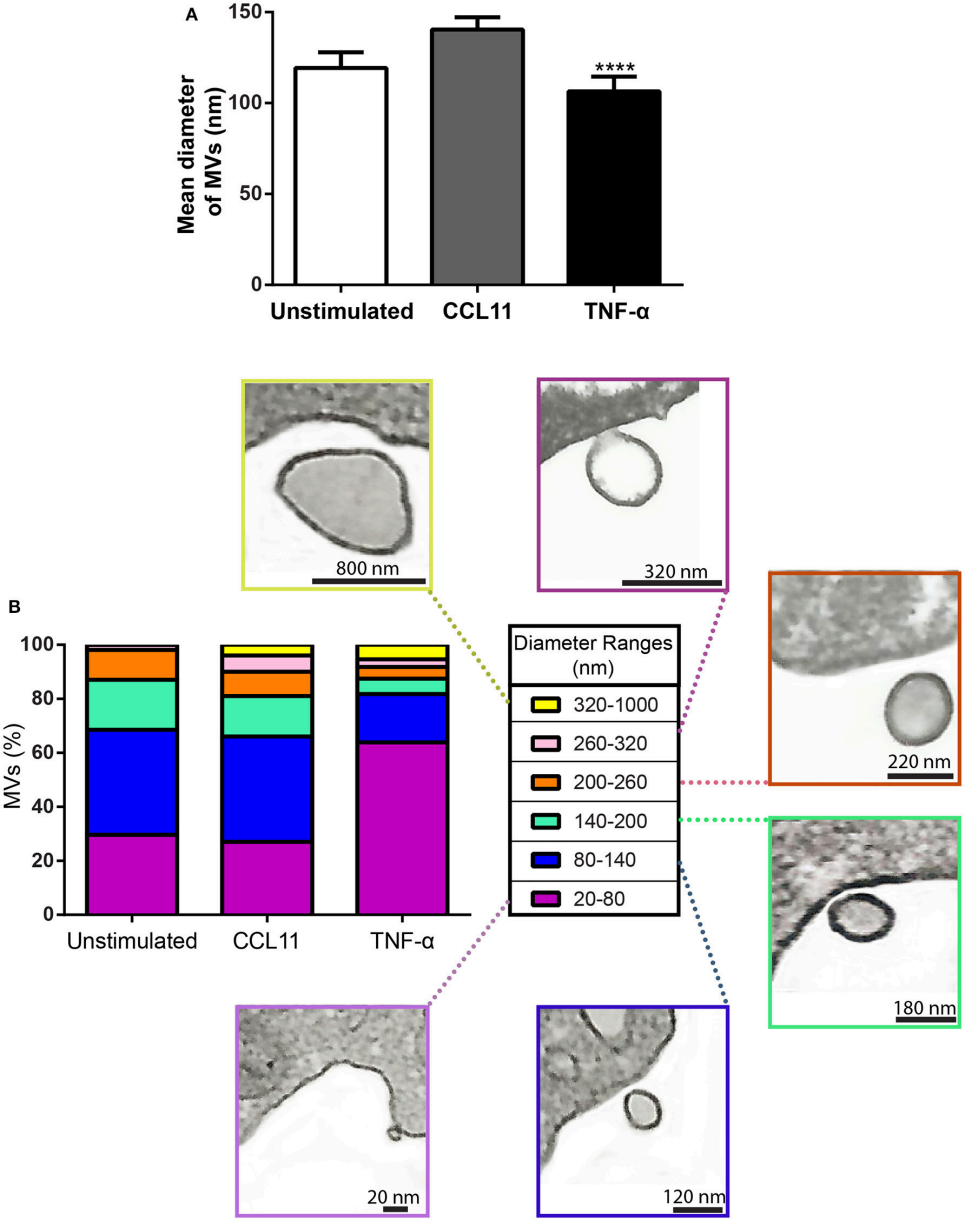
**Diameter of MVs produced by unstimulated and stimulated human eosinophils. (A)** Determination of mean diameter of MVs. Significant decrease in the diameters of released MVs occurred after stimulation with TNF-α compared to both unstimulated and CCL11-stimulated groups (^****^P < 0.0001). **(B)** The percentages of MVs per diameter ranges are shown. Representative electron micrographs of MVs are seen within each diameter range. Diameters of MVs were measured using *Image J software* and grouped in different ranges (20–100, 100–200, 200–300, 300–1000 nm). These analyses were done in clear cross-cell sections exhibiting the entire eosinophil cell profile (*n* = 110 cells), intact plasma membranes, and nuclei.

In addition to quantification studies, we also established the average size of MVs to be 119.30 ± 8.61 nm (mean ± SEM) in diameter in control cells and 140.40 ± 6.80 and 106.50 ± 6.07 (mean ± SEM) nm in CCL11 and TNF-α, respectively (Figure 6A). Considering all conditions, eosinophil EV diameters varied from 20 to 1000 nm, with most MVs showing diameters between 20 and 200 nm (Figure 6B). MVs released in response to TNF-α were significantly smaller compared to those released after CCL11 stimulation and by unstimulated cells (*P* < 0.0001; Figures 6A,B).

**Figure 7:**
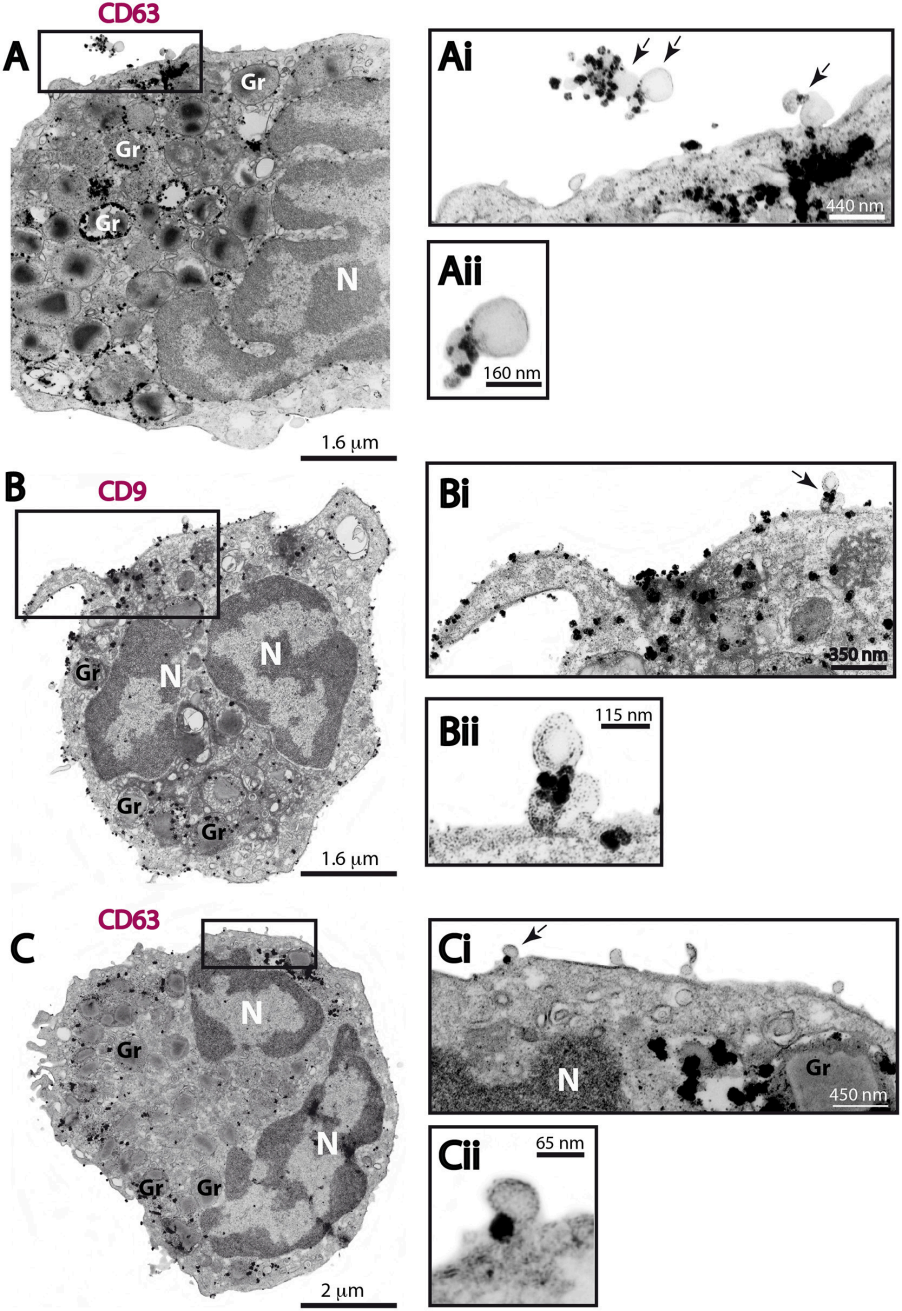
**CD63 and CD9 immunolabeling of MVs by immunonanogold EM**. **(A–C)** Representative electron micrographs of stimulated human eosinophils showing the entire cell profile after CD63 **(A,C)** or CD9 **(B)** immunolabeling. Note that while CD63 is consistently found intracellularly in association with secretory granules (Gr) as seen in **(A)** and **(C)**, pools of CD9 are more detectable at the eosinophil surface as observed in **(B)**. **(Ai, Aii, Bi, Bii, Ci, Cii)** CD63 and CD9-positive MVs (arrows) are seen in higher magnification in the boxed areas. Note, that not all MVs were labeled. Eosinophils were isolated from the peripheral blood by negative selection, stimulated for 1 h with CCL11 **(A,B)** or TNF-α **(C)**, immediately fixed in suspension and processed for immunonanogold EM.

Next, we investigated if the MVs produced by human eosinophils expressed CD63 or CD9. Ultrastructural immunolabeling for these tetraspanins were achieved with pre-embedding immunonanogold EM, a technique that has been used by us to ascertain precise localization of cytokines, immune cell signaling molecules and tetraspanins in leukocytes (Melo et al., 2014). In previous works, we have defined the ultrastructural pattern of immunolabeling for these tetraspanins in human eosinophils (Akuthota et al., 2012; Carmo et al., 2016). While CD63 is consistently found intracellularly in association with granules undergoing losses of their contents and large vesicular carriers (Carmo et al., 2016), pools of CD9 are more detectable at the eosinophil surface (Akuthota et al., 2012). In the present work, ultrastructural immunolabeling for CD63 and CD9 at MVs was investigated for the first time. All groups showed clear immunonanogold labeling for both CD63 (Figures 7A,C) and CD9 (Figure 7B). However, not all MVs were positive (see, for example Figure 7Ci). In both unstimulated and stimulated cells, immunolabeling for CD9 and CD63 were found in around 50 and 15% of the MVs, respectively, regardless of the stimulation condition.

Control cells, from all conditions, in which the primary antibody was omitted or replaced by an irrelevant antibody were negative (Supplementary Figure 4).

### Annexin V staining of stimulated human eosinophils

**Figure 8:**
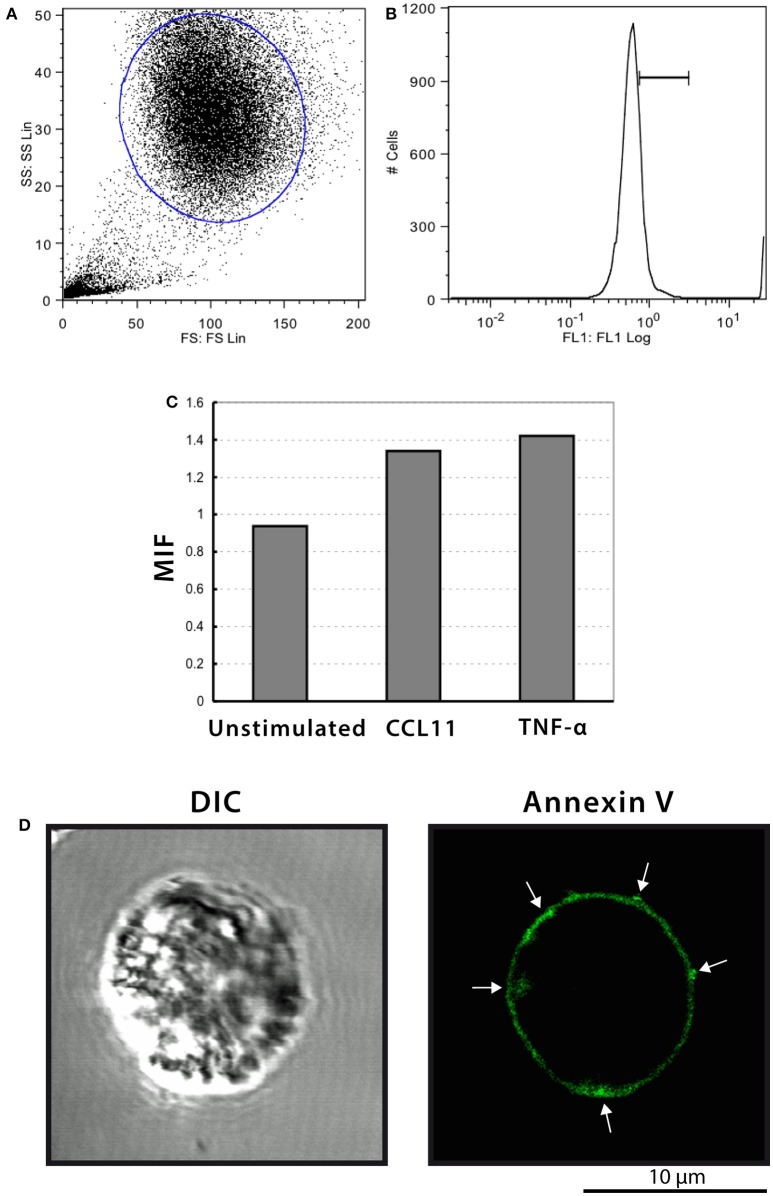
**Annexin-V staining of human eosinophils. (A–C)** Flow cytometric analyses show higher annexin-V intensities in CCL11 and TNF-α-stimulated compared to unstimulated cells. **(D)** Representative confocal microscopic image from a CCL11-stimulated eosinophil reveal cell surface distribution of annexin-V. Arrows indicate suggestive images of MV formation. Eosinophils were isolated from the peripheral blood by negative selection, stimulated for 1 h with CCL11 or TNF-α and stained with annexin-V.

It is recognized that phosphatidylserine is relocated to the outer membrane leaflet at sites on the cell surface where MV shedding occurs (reviewed in Hugel et al., 2005; Muralidharan-Chari et al., 2010). Then, we next stained eosinophils with annexin-V-FITC and samples were analyzed by both flow cytometry and confocal microscopy. Intact eosinophils were gated and their representative histogram is shown in Figures 8A,B, respectively. The histogram depicted unimodal distribution indicating that most cells were negatively stained by annexin-V. However, higher annexin-V intensities were observed in CCL11 and TNF-α stimulated compared to unstimulated eosinophils (Figure 8C). Confocal microscopy analyses showed cell surface distribution of annexin-V with suggestive images of MV formation in a population of stimulated cells (Figure 8D). The absence of noticeable TUNEL positive cells in CCL11 and TNF-α stimulated cells (Supplementary Figure 5) as well as by TEM indicated these are not apoptotic bodies.

## Discussion

The production of EVs during immune responses has increasingly been demonstrated. These vesicles released by cells from the immune system have been characterized as a new mechanism of cell-to-cell communication and emerged as potential mediators of the cell immune effects (reviewed in Buzas et al., 2014; Colombo et al., 2014; Robbins and Morelli, 2014; Greening et al., 2015). Here, we identified, for the first time, that human eosinophils release MVs in physiological conditions and that these cells respond to the chemokine CCL11 and the cytokine TNF-α stimuli with increased formation of these plasma membrane-derived vesicles. We thus recognized that, in addition to the secretory processes largely described for human eosinophils (PMD, classical exocytosis and cytolysis), these cells also have the competence to secrete MVs and that these vesicles likely underlie eosinophil immune responses.

The cell biology of EVs is still poorly understood. To comprehend the origin of the different populations of these secreted membrane-bound vesicles and their functional significance, a better knowledge of their mechanisms of biogenesis and secretion is still needed (Colombo et al., 2014). Here we provided a comprehensive evaluation of EVs secreted by human eosinophils using TEM, which is considered the gold standard for EV visualization (Lawson et al., 2016). Our present work highlights important aspects to the maturing field of EV research. First, it is clear that EVs cannot be defined on the basis of their size range as exosomes or MVs since these classes of EVs have overlapping sizes. In general, exosomes are considered to be smaller (~50–100 nm) than MVs (reviewed in van der Pol et al., 2012; Lawson et al., 2016), but we found that MVs from eosinophils can be as small as 20 nm (range of 20–1000 nm), with most MVs measuring 20–200 nm. In the literature, MVs have been reported as a heterogeneous population in size up to 2000 nm (reviewed in Buzas et al., 2014; Schwab et al., 2015; Lawson et al., 2016). Because our electron microscopic analyses were done on a population of MVs clearly seen at cell surface (nascent MVs), when the cells were still in suspension, we believe that the observed diameter range is more precise than those established on isolated vesicles. Indeed, because of the small size of MVs, a considerable portion of them may be below the detection range of conventional detection methods (van der Pol et al., 2012). Moreover, mechanical disruption of the cells/tissues can interfere with the EV purity, since intracellular vesicles might also be isolated during the process (Lötvall et al., 2014). This is particularly concerning for human eosinophils, which have a well-characterized intracellular morphologically distinct vesicular system termed eosinophil sombrero vesicles (EoSVs; 150–300 nm diameter) that can be isolated and maintain their integrity even after cell cytolysis (Melo et al., 2005b, 2008b, 2009; Saffari et al., 2014). Second, our findings demonstrate, for the first time, that, depending on the influence of external factors/stimuli, the sizes of MVs can vary (Figure 6). Thus, MVs released from TNF-α-stimulated eosinophils exhibited smaller size compared to CCL11-stimulated cells (Figure 6). This might be explained by the rapid production of these membranous structures after stimulation, which may be affecting membrane replenishment and dynamics required for vesicle formation.

The present work also raises discussion on an important point of the EV biology: the use of appropriate markers for EVs released by non-immune and immune cells. In general, the tetraspanins CD63 and CD9 have been proposed as “universal” EV markers and exosomes have been described as highly enriched in these molecules (reviewed in Andreu and Yáñez-Mó, 2014). However, the presence of CD63 and CD9 in plasma membrane-derived MVs has been much less studied, regardless of cell type. Few studies have currently assumed that MVs express tetraspanins (Crescitelli et al., 2013). Here, we provide direct evidence for both CD63 and CD9 localization on MVs secreted by human eosinophils (Figure 7). Our immunonanogold EM findings clearly showed CD63 and CD9 labeling associated with the delimiting membrane of MVs (Figure 7). However, in accord with our nanoscale flow cytometry results (Figure 2), immunoEM also revealed that not all MVs were labeled (see, for example, Figure 7Ci), with ~50 and ~15% of these vesicles labeled for CD9 and CD63, respectively, regardless of whether cells were stimulated or not. CD63 was not detected by flow cytometry, possibly because of the very small size and/or low proportion of this CD63-positive EV population. Therefore, CD9, a tetraspanin largely found at the surface of human eosinophils (Akuthota et al., 2012) might be a better marker than CD63 for MVs released by human eosinophils.

At first view it seems unexpected to have undetectable levels (as observed by flow cytomety) or low labeling (as seen by immunoEM) for CD63 on MVs. However, we have demonstrated in a recent study by different approaches that while CD63 is observed at the eosinophil's cell surface after stimulation with both CCL11 and TNF-α, a robust pool of CD63 remains in the cytoplasm in association with secretory granules and EoSVs, with no detectable difference in the CD63 total content when unstimulated and stimulated cells were compared (Carmo et al., 2016). This means that CD63 is present, as a preformed pool within eosinophils and that most of this internal CD63 pool is not completely externalized in response to stimulation (Carmo et al., 2016). Accordingly, a small proportion of secreted MVs showed immunolabeling for this tetraspanin. Moreover, our comprehensive EM analyses demonstrated that eosinophils release MVs and not typical exosomes in response to stimulation with CCL11 or TNF-α. Although MVBs were observed in the eosinophil cytoplasm during the present EM analyses, there was no ultrastructural evidence for exosome secretion. Our data are in part in accord with a work showing that stimulation of human eosinophils with CCL11 does not appear to increase secretion of CD63-positive exosomes (Mazzeo et al., 2015). On the other hand, these authors found that stimulation with interferon-gamma (INF-γ) induced exosome secretion by these cells (Mazzeo et al., 2015).

Here, induction of EV release was achieved with TNF-α and CCL11. TNF-α is a potent cytokine that mediates inflammatory responses and innate immunity (reviewed in Sabio and Davis, 2014). Stimulation of human eosinophils with TNF-α induces secretion of IL-4, IL-6 and INF-γ (Spencer et al., 2009). TNF-α is also central for INF-γ-induced secretion of Th1-type chemokines and to enhance IL-4-induced secretion of Th2-type chemokines by human eosinophils (Liu et al., 2007). We recently demonstrated that this stimulus leads to a secretory process characterized by fusion of eosinophil secretory granules (classical exocytosis) and extensive release of granule contents while CCL11 elicits a progressive and more subtle release of specific products stored in secretory granules (piecemeal degranulation; Carmo et al., 2016). For example, CCL11 stimulation of human eosinophils elicits specific release of IL-4 (Bandeira-Melo et al., 2001). Our present data showed that the differential secretory/immune responses induced by these two stimuli trigger differential rates of EV release. Higher numbers of MVs were detected in eosinophils in response to TNF-α compared to CCL11 stimulation. Thus, MVs released by human eosinophils may potentially carry different cargos and mediate different effects on other cells, depending on the stimulus/pathological condition. We can speculate that eosinophil MVs might be acting as potential mediators of immune responses. The release of them at inflammatory sites in tissues and/or in biological fluids, including peripheral blood, may enable *in situ* and/or long-distance transfer of bioactive molecules such as cytokines. These molecules may influence target cells by activating cell receptors with vital roles in inflammation. In fact, the implication of MVs in inflammation has been documented. For example, the presence of interleukin-1β was detected in MVs shedding from the plasma membrane of activates monocytes (MacKenzie et al., 2001). However, the identification of molecular cargos within these eosinophil-released MVs awaits further investigation to get insights into their functional roles.

Taken together, our findings identify, for the first time, that human eosinophils secrete MVs during physiological conditions and that the release of these vesicles is increased in response to both CCL11 and TNF-α. Given the potential of EVs as mediators of immune responses, our results open new venues to understand how these vesicles function to regulate eosinophil-mediated immunity and if they can be used as biomarkers for eosinophil-associated disorders.

## Author contributions

RCNM and PA provided the study conception and design. RCNM, PA, and PW provided study guidance, mentorship, and critical editing of the manuscript. LC, KB, ROM, TS, JG, KC, SU, RCNM performed experiments, acquired, and analyzed the data. PA, IG, LC, KB, TS, JG, SU, PW, RCNM interpreted data. VT, JT, VC, and SU performed flow cytometric analyses. RCNM and PA prepared the manuscript. All authors contributed in part to writing and editing the manuscript and approved the final version.

### Conflict of interest statement

The authors declare that the research was conducted in the absence of any commercial or financial relationships that could be construed as a potential conflict of interest. The reviewer MP and handling Editor declared their shared affiliation, and the handling Editor states that the process nevertheless met the standards of a fair and objective review.
