# Supplementary material for: Extracellular Microvesicle Production by Human Eosinophils Activated by “Inflammatory” Stimuli
Source: Front Cell Dev Biol. 2016 Oct 27;4:117. doi: 10.3389/fcell.2016.00117 (PMC5081571; doi:10.3389/fcell.2016.00117)
Supplement: Supplementary file 1 [file Image1.pdf]

Supplementary Material

# Extracellular microvesicle production by human eosinophils activated by "inflammatory" stimuli

**Praveen Akuthota, Lívia A. S. Carmo, Kennedy Bonjour, Ryann O. Murphy, Thiago P. Silva, Juliana P. Gamalier, Kelsey L. Capron, John Tigges, Vasilis Toxavidis, Virginia Camacho, Ionita Ghiran, Shigeharu Ueki, Peter F. Weller and Rossana C. N. Melo***

*** Correspondence:** Rossana C. N. Melo - rossana.melo@ufjf.edu.br

## 1 Supplementary Figures

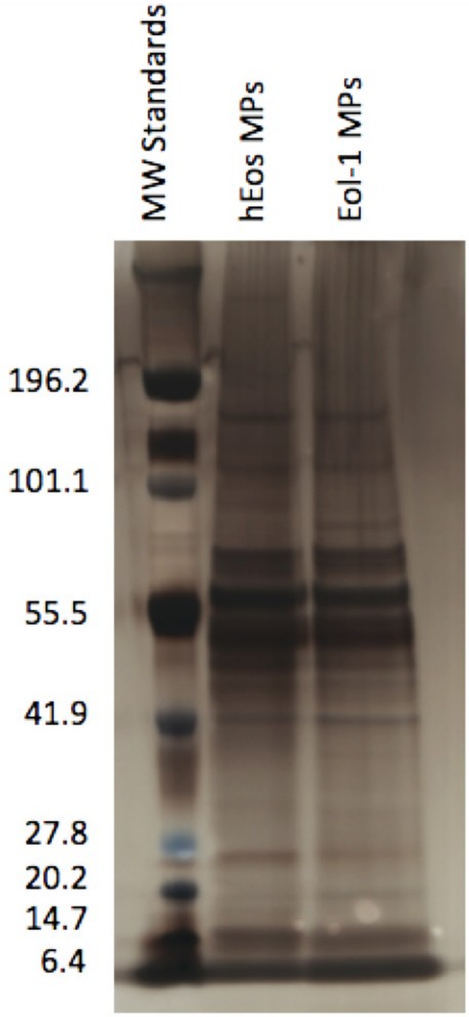


**Supplementary Figure 1. Polyacrylamide gel electrophoresis of isolated EVs followed by silver stain.** Samples from primary eosinophil cultures (hEos) and eosinophilic leukemia cell line (Eol-1) cultures were assayed. A band at 25 kD, the expected molecular weight for CD9, was observed in both samples. Molecular Weight (MW) standards are given in kD.

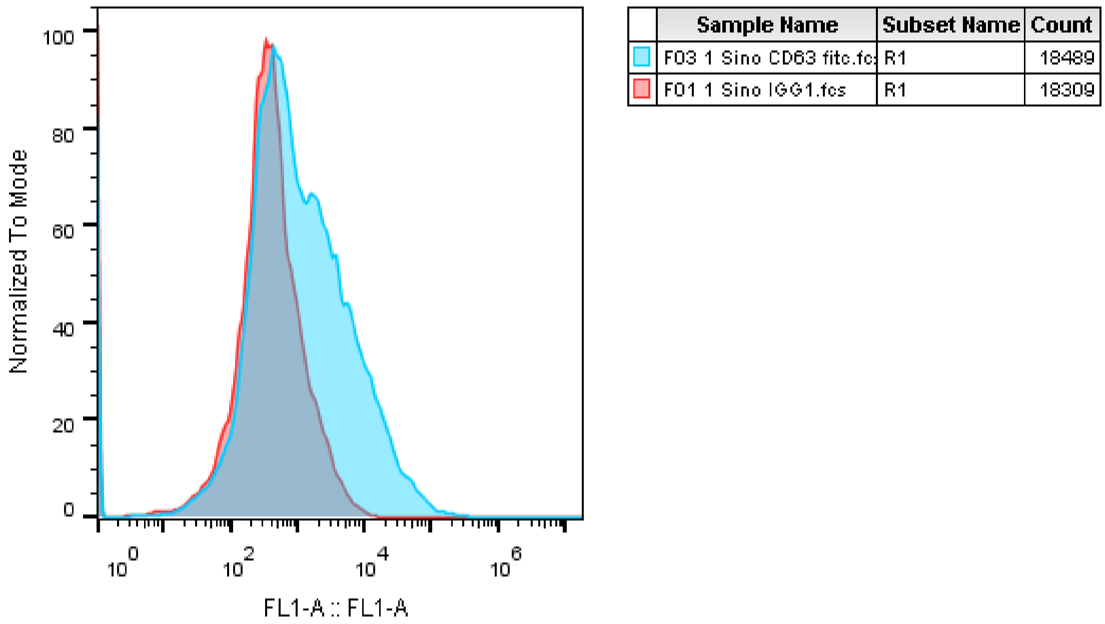


**Supplementary Figure 2. Flow cytometry analysis for CD63 of a mixed granulocyte population of eosinophils and neutrophils was performed as a positive control**. CD63 histogram is depicted in blue and IgG1 isotype control is depicted in red.

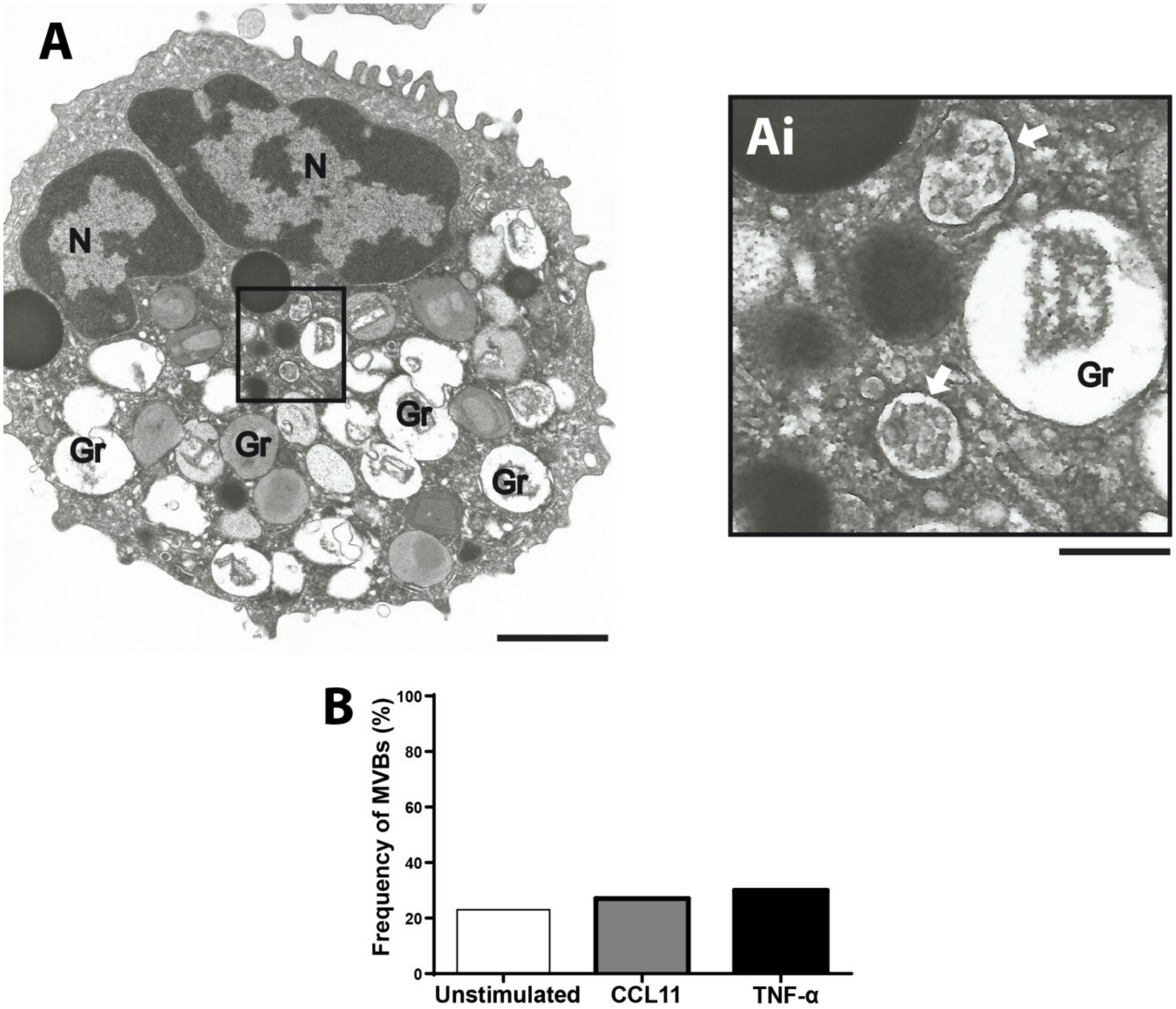


**Supplementary Figure 3. Transmission electron microscopy (TEM) analyses of multivesicular bodies (MVBs) in human eosinophils.** (A) A representative electron micrograph from a CCL11-stimulated eosinophil shows MVBs in the cell cytoplasm. In (Ai), the typical morphology of MVBs is seen in high magnification (arrows). (B) Frequency of cells with cytoplasmic MVBs in unstimulated and CCL11 or TNF- α-stimulated eosinophils. A total of 110 electron micrographs were analyzed and the number of MVBs determined. Eosinophils were isolated from the peripheral blood by negative selection, stimulated for one hour and processed for conventional TEM. Gr, secretory granule; N, nucleus. Scale bar: 1.0 μm (A); 0.3μm (Ai) .

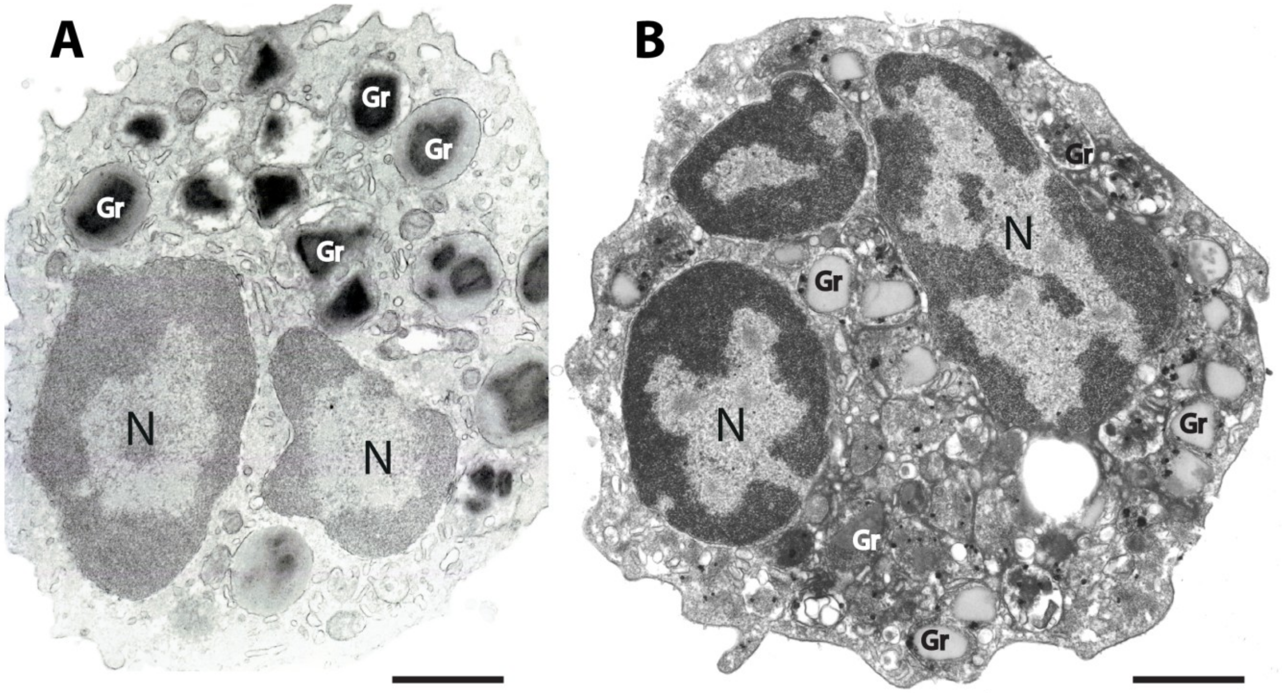


**Supplementary Figure 4. Representative electron micrographs from human eosinophils in which the primary antibody was replaced by an irrelevant antibody show negative or negligible labeling for CD63 (A) or CD9 (B).** Eosinophils from healthy donors were isolated by negative selection, kept in medium (A) or stimulated with CCL11 (B) for one hour and processed for immunonanogold electron microscopy. Gr, secretory granule; N, nucleus. Scale bar: 1.2 µm (A); 1.0 µm (B).

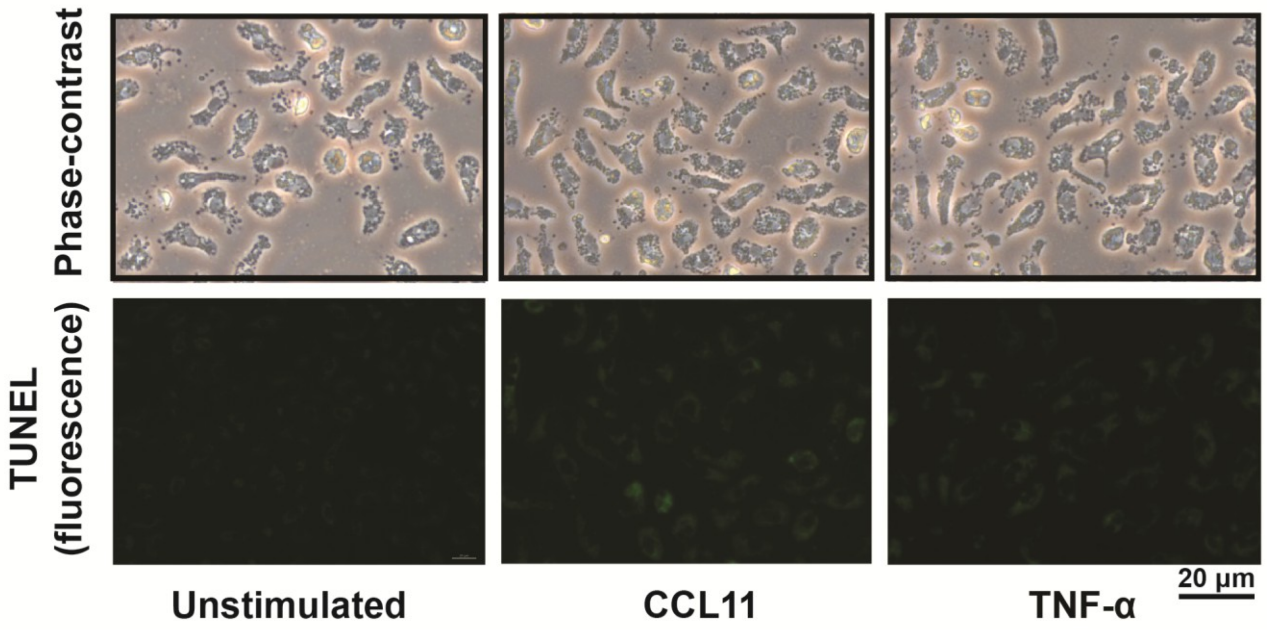


**Supplementary Figure 5. TUNEL analyses of human eosinophils show absence or negligible staining for apoptosis.** Phase-contrast (upper panels) and fluorescence (bottom panels) microscopy of identical fields of eosinophil stimulated or not for one hour with CCL11 or TNF-α.Eosinophils were isolated from the peripheral blood by negative selection, stimulated, fixed in 4% paraformaldehyde and stained with apoptosis TUNEL kit.
